# Numerical study on infill fracturing of mature wells under free-drainage boundary conditions

**DOI:** 10.1371/journal.pone.0354099

**Published:** 2026-10-27

**Authors:** Xingwen Jiang, Ziyuan Cong, Hubuqin Hubuqin, Genbo Peng, Yuwei Li

**Affiliations:** 1 StateKey Laboratory of Continental Shale Oil, Northeast Petroleum University, Daqing, Heilongjiang, China; 2 School of Environment, Liaoning University, Shenyang, Liaoning, China; The University of Hong Kong, HONG KONG

## Abstract

Infill fracturing of mature wells involves re-fracturing within previously stimulated clusters, targeting unstimulated regions of the parent well's fracture network. This strategy sustainably enhances hydrocarbon recovery from depleted reservoirs without the environmental footprint of new drilling. Establishing a numerical simulation model for this process is essential for optimization. In this study, a numerical model for mature well infill fracturing was developed based on the extended finite element method and a free-drainage boundary module. The results indicate that prior production from mature wells primarily affects the fracture length and curvature in the infill wells. As infill fracturing-induced fractures propagate toward the far-field region, they are significantly influenced by the heterogeneous pore pressure distribution in the reservoir. This leads to a pronounced increase in fracture curvature. Compared with conventional fracturing, the curvature of fractures under infill fracturing is approximately 30% higher within the same simulation time, while the fracture length is notably shorter. With the increase in production time, fractures in infill wells tend to exhibit greater tortuosity. This enhanced complexity allows for more effective contact between the fractures and the reservoir, thereby improving the production potential of the infill wells to some extent.

## 1. Introduction

Unconventional resources such as shale gas and tight oil offer cleaner-burning alternatives compared to conventional fossil fuels, with lower carbon intensity and reduced environmental impact per unit of energy produced, making their sustainable development crucial for the energy transition. Unconventional oil and gas reservoirs are typically characterized by low porosity and low permeability. Conventional development methods are associated with high operational costs and poor production performance, making them insufficient for meeting the demands of commercial exploitation [1,2]. Volume fracturing technology addresses this challenge by injecting large volumes of high-pressure fluid into the reservoir to forcibly open the rock matrix. The resulting complex fracture network significantly enhances hydrocarbon migration pathways within the pore system and has become a key technique for the efficient development of unconventional resources [3,4]. However, field production practices have revealed that many fractured wells suffer from rapid production decline and short periods of stable output. In some cases, stable production can only be maintained for a few months [5]. The primary reason lies in the limitations of both the fracturing technology and reservoir characteristics, which result in suboptimal initial fracturing performance. The induced fractures often fail to effectively connect with the far-field reservoir, restricting hydrocarbon recovery to the near-wellbore zone and ultimately limiting the production capacity of fractured wells [6]. From a sustainability perspective, maximizing the recovery factor from existing wells is preferred over drilling new wells, as it minimizes surface disturbance, reduces the consumption of freshwater and proppants per unit of energy produced, and lowers greenhouse gas emissions associated with drilling operations.

The infill fracturing technique for mature wells involves re-fracturing within a cluster of previously fractured wells—commonly referred to as “parent wells” The newly fractured wells, also known as “child wells” are designed to target the unstimulated zones between or beyond the fracture networks of the parent wells, thereby enhancing the overall development efficiency of unconventional reservoirs [7,8]. At present, infill fracturing of mature wells has been widely applied in countries such as the United States, Canada, and China [9]. The implementation of infill fracturing in mature wells requires careful consideration of both the timing of the infill operation and the associated fracturing parameters [10]. Developing a scientifically sound and effective numerical model for mature well infill fracturing is essential for optimizing design parameters and provides critical technical support and theoretical guidance for the success of such operations.

Relevant studies have shown that infill fracturing in mature wells does not necessarily lead to increased production capacity [11]. Miller et al.[12] reported that, in the Bakken Basin of the United States, 64% of parent wells experienced production decline following infill fracturing. Similar trends were observed in the Haynesville and Woodford Basins, where 58% and 64% of parent wells, respectively, showed reduced productivity after infill operations. Infill fracturing of child wells can help replenish reservoir energy and potentially reactivate pre-existing fractures in parent wells, thereby enhancing the production performance of both parent and child wells. However, hydraulic fractures from child wells may also compete with those of the parent wells, adversely affecting their productivity [13]. The complex interactions between parent and child wells make it challenging to predict the fracture propagation trajectories during infill operations. To address these challenges, numerous numerical studies have been conducted on mature well infill fracturing [14,15]. Guo et al.[16] developed a numerical model for mature well infill fracturing using the finite element method to investigate the effects of various fracture and rock mechanical parameters on fracture propagation. Ren et al.[17] employed the discrete element method to construct a numerical model of secondary fracturing that incorporates natural fractures, further analyzing the morphology of child well fractures under the influence of weak natural planes. Safari et al.[18] proposed a hybrid numerical approach combining the boundary element method and finite difference method to study mature well infill fracturing, revealing that fracturing in parent wells alters the original in-situ stress field, causing hydraulic fracture deflection in the infill wells. Yang et al.[19] proposed a coupled finite difference–discrete element model to analyze the mechanical mechanisms underlying the asymmetric propagation of hydraulic fractures in infill wells under dynamically evolving in-situ stress conditions. During the depletion phase of reservoir development, the gradual reduction in pore pressure leads to significant changes in the stress state of the formation, which in turn has a profound impact on the fracture propagation behavior of subsequently fractured child wells. The stress evolution induced by long-term production from mature wells represents a typical fluid–solid coupling problem. Previous studies have made substantial theoretical and numerical progress in this field [20,21]. However, the theoretical framework for understanding hydraulic fracture propagation under stress disturbances caused by prior production remains incomplete. The evolutionary patterns and dominant controlling factors of fracture propagation in such contexts require further in-depth investigation.

The optimization of construction parameters for mature well infill fracturing is a complex process that requires maximizing the productivity of child wells while minimizing their adverse impact on parent well performance. This involves addressing several critical issues, including reservoir condition changes during parent well production, non-uniform fracture propagation, and well-to-well interference between infill and existing wells. Key operational parameters such as infill timing, perforation orientation, and injection rate must be carefully considered [22,23]. To address the above issues, this study develops a fracture propagation model based on the cohesive traction–separation law integrated with the extended finite element method (XFEM), enabling the simulation of asymmetric fracture growth under inter-well interference. A free-drainage boundary condition is incorporated to simulate fluid outflow from the parent well fractures, allowing for a more realistic representation of pore pressure evolution in the reservoir at different production times. Using this numerical model, the influence of key engineering parameters on the performance of infill fracturing is investigated, providing theoretical guidance for the design and optimization of mature well infill fracturing operations.

## 2. Modeling and method

The infill fracturing model for mature wells differs from conventional hydraulic fracturing models. In addition to simulating the cooperative propagation of multiple fractures [24], it must also accurately capture the dynamic evolution of pore pressure during parent well production. Accordingly, the numerical model developed in this study consists of three integrated modules: a solid deformation and fracture module, a fluid flow module, and a production-driven free-drainage boundary module.

### 2.1 Solid deformation and fracture module

The constitutive behavior of the rock matrix is primarily modeled as elastic and is calculated using the generalized Hooke’s law [25,26]. The corresponding formulation is expressed as follows:


$$\begin{array}{c} \sigma =D^{\text{el}}\varepsilon^{\text{el}} \end{array}$$ (1)

$$\begin{array}{c} D^{\text{el}}=\left[\begin{array}{cccccc} @cccccc@\lambda +2G & \lambda & \lambda & 0 & 0 & 0\\ \lambda & \lambda +2G & \lambda & 0 & 0 & 0\\ \lambda & \lambda & \lambda +2G & 0 & 0 & 0\\ 0 & 0 & 0 & G & 0 & 0\\ 0 & 0 & 0 & 0 & G & 0\\ 0 & 0 & 0 & 0 & 0 & G \end{array}\right] \end{array}$$ (2)

$$\begin{array}{c} G=\frac{E}{2(1+\nu )} \end{array}$$ (3)

$$\begin{array}{c} \lambda =\frac{E\nu }{\left(1+\nu )(1-2\nu \right)} \end{array}$$ (4)

where *σ* is the total stress, Pa; $\varepsilon^{\text{el}}$ is the total elastic strain (dimensionless); $D^{\text{el}}$ is the elastic stiffness tensor; *λ* is the Lamé constant, Pa; *G* is the shear modulus, Pa; *E* is the Young’s modulus, Pa; *v* is the Poisson’s ratio.

The principle of effective stress is employed to couple the pore pressure with the stress field, expressed as:


$$\begin{array}{c} \sigma^{\prime }=\sigma -\alpha p_{p} \end{array}$$ (5)

where: $\sigma^{\prime }$ is the effective stress, $p_{p}$ is the pore pressure, α is the Biot coefficient.

Rock fracture is modeled using the extended finite element method (XFEM), which offers the advantage of mesh flexibility—fracture geometry does not need to conform to the mesh, allowing for the use of structured grids. This significantly improves both convergence and computational efficiency. The mesh discretization is illustrated in Fig 1. The fundamental theory of XFEM has been detailed in previous studies [27,28] and will not be repeated here. In this study, the constitutive behavior of XFEM elements is governed by a traction–separation (S–T) model, whose constitutive relationship is shown in Fig 2.

**Fig 1:**
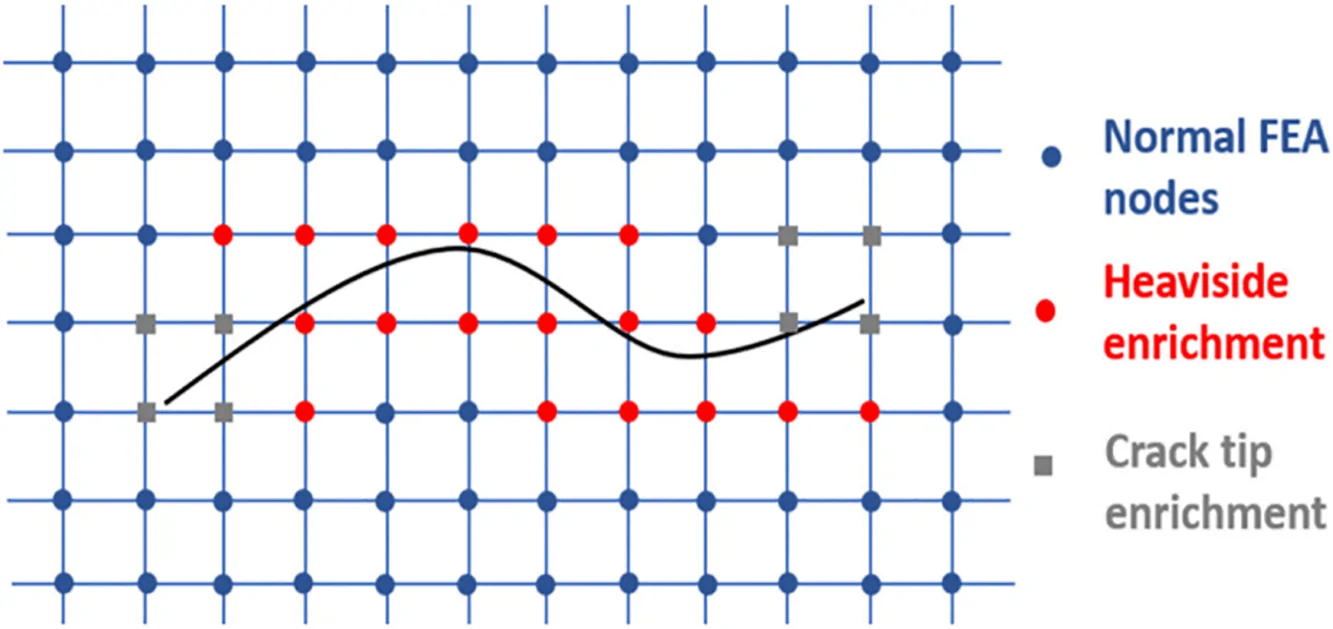
Schematic diagram of XFEM discretization [29].

**Fig 2:**
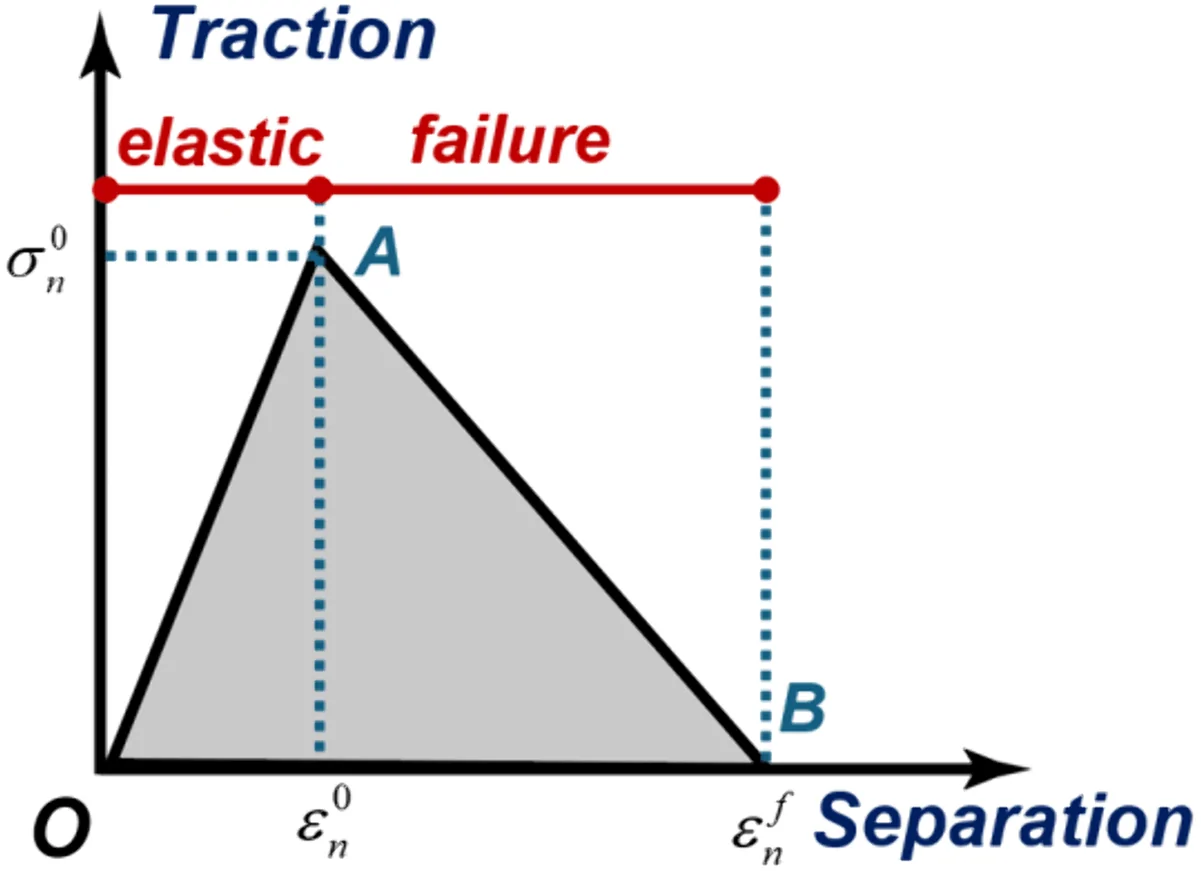
Constitutive of the traction–separation model.

The traction–separation (S–T) model consists of two stages: (1) An undamaged stage with constant stiffness (segment OA); (2) A damage evolution stage characterized by progressive stiffness degradation (segment AB). A damage factor *D*, ranging from 0 to 1, is introduced. In the undamaged stage, the stress–displacement relationship is linear, the element stiffness remains unchanged, and *D* is constantly zero. When the total relative displacement between the top and bottom surfaces of a cohesive element reaches the initial damage displacement $\varepsilon_{n}^{\text{0}}$, the element enters the damage evolution stage (segment AB).

The constitutive relationship of the S–T model can be expressed by the following equations:


$$\begin{array}{c} t_{\text{n}}=M_{i}\varepsilon_{\text{n}} \end{array}$$ (6)

where $t_{\text{n}}$ is the traction, Pa; $t_{\text{n}}$ is the separation strain; and Mi is the element stiffness, Pa; equal to the elastic modulus in the undamaged stage.

In the damage evolution stage, the element stiffness *M*_*i*_ gradually decreases, while the damage factor *D* increases from 0. The calculation formula is as follows:


$$\begin{array}{c} \left\{ \begin{array}{c} @l@M_{i}=M(1-Dnonumber\\ D=\frac{d_{m}^{f}(d_{m}^{max}-d_{m}^{\text{0}})}{d_{m}^{max}(d_{m}^{f}-d_{m}^{\text{0}})} \end{array}\right. \end{array}$$ (7)

where *M* is the element stiffness in the undamaged stage, *M*_*i*_ is the element stiffness in the damaged stage, *D* is the damage variable; $d_{m}^{max}$ is the maximum displacement of the element, m; $d_{m}^{f}$ is the current opening displacement, m; $d_{m}^{\text{0}}$ is the displacement at the onset of damage, m.

Regarding the value of $\varepsilon_{n}^{\text{0}}$, this study adopts the maximum nominal stress criterion, with the calculation formula given as:


$$\begin{array}{c} {\left\{\frac{\langle \sigma_{n}\rangle }{\sigma_{n}^{\text{0}}},\frac{\sigma_{s}}{\sigma_{s}^{\text{0}}},\frac{\sigma_{t}}{\sigma_{t}^{\text{0}}}\right\}}_{max}=1 \end{array}$$ (8)

where $\sigma_{n}^{\text{0}}$, $\sigma_{s}^{\text{0}}$, $\sigma_{t}^{\text{0}}$ are the normal and the first and second tangential peak stresses, Pa; respectively; ⟨⟩ denotes that the element only bears tensile stress.

### 2.2 Fluid flow module

Assuming no relative slip between the fracture walls and a uniform fluid pressure drop within the fracture, and neglecting the normal flow of fracturing fluid across adjacent fracture surfaces, the lubrication equation can be expressed as follows [30]:


$$\begin{array}{c} \frac{dp}{dx}\text{=}\frac{12q\mu }{w^{\text{3}}h} \end{array}$$ (9)

where *p* is the fluid pressure within the fracture, MPa; *x* is the fracture length, m; *q* is the injection rate, m³/min; *μ* is the fracturing fluid viscosity, mPa·s; *w* is the fracture width (i.e., the normal displacement from the rock deformation equation), m; *h* is the reservoir thickness, m.

The mass conservation equation for fluid within the fracture is given as follows [31]:


$$\begin{array}{c} \frac{\partial q\left(x,t\right)}{\partial x}-q_{L}\left(x,t\right)=\frac{\partial w\left(x,t\right)}{\partial t} \end{array}$$ (10)

where *q*_L_ is the fluid leak-off rate, m^2^/min.

The fluid leak-off rate can be calculated using the Carter leak-off model [32]:


$$\begin{array}{c} q_{L}\left(x,t\right)=\frac{2hC_{L}}{\sqrt{t-\tau \left(x\right)}} \end{array}$$ (11)

where *C*_L_ is the leak-off coefficient, m/min^0.5^; *t* is the total injection time, min; and *τ* is the time when the fracture element begins to leak off, min.

The total injection rate for multiple fractures is the sum of the injection rates for each individual fracture, expressed as:


$$\begin{array}{c} Q_{T}=\sum_{i=1}^{m}Q_{i} \end{array}$$ (12)

According to Kirchhoff’s second law, the pressure in the wellbore satisfies:


$$\begin{array}{c} p_{\text{0}}=p_{w,i}+p_{f,i}+p_{cf,i}\text{\textasciitilde{}}(i=1,2,3,...,m) \end{array}$$ (13)

where *p*_0_ is the fluid pressure at the heel of the horizontal wellbore, MPa; *p*_*w,i*_ is the fluid pressure at the entrance of the *i* th fracture, MPa; *p*_*pf,i*_ is the perforation friction for the *i* th fracture, MPa; and *p*_*cf,I*_ is the wellbore friction for the *i* th fracture, MPa.

The wellbore friction can be calculated by Equation (14):


$$\begin{array}{c} p_{pf,i}=\frac{0.2369\rho_{s}}{n_{p,i}^{\text{2}}d_{p,i}^{\text{4}}C_{d,i}^{\text{2}}}Q_{i}^{\text{2}} \end{array}$$ (14)

where *p*_*pf*_ is the perforation friction, Pa; *ρ*_*s*_ is the fracturing fluid density, kg/m³; *n*_*p,i*_ is the number of perforation holes for the *i* th fracture, dimensionless; *d*_*p,i*_ is the diameter of the perforation holes for the *i* th fracture, m; and C_*d,i*_ is the discharge coefficient correction factor for the perforation holes of the *i* th fracture, m.


$$\begin{array}{c} p_{cf,i}=C_{cf}\sum_{j=1}^{i}(x_{j}-x_{j-1})Q_{w,j} \end{array}$$ (15)

where *p*_*cf*_ is the wellbore friction, Pa; *C*_*cf*_ is the friction coefficient, Pa·s/m^4^; *x*_*j*_ is the distance from fracture *j* to the heel of the horizontal wellbore, m; and *Q*_*w,j*_ is the remaining fluid flow rate after passing through fracture *j*, m^3^/min.

### 2.3 Drainage boundary module

In numerical simulations, the production process must satisfy the following requirements: (1) the seepage fluid within the model must be able to flow out across specific boundaries; (2) there is a pore pressure threshold at the wellbore surface, below which no fluid flows out and the flow rate is zero; and (3) when the pore pressure exceeds this threshold, the fluid flows out of the model unimpeded, thereby reducing the wellbore pore pressure. The free drainage boundary perfectly meets these requirements and is theoretically feasible.

In this study, the hydraulic fractures in the parent wells are defined as drainage boundaries that allow reservoir fluid to flow out, enabling simultaneous production from the parent well and infill fracturing in the new well. The fluid outflow rate at the drainage boundary is assumed to be proportional to the local pore pressure, as described in Equation (16):


$$\begin{array}{c} v_{\text{n}}=k_{\text{s}}(u_{\text{w}}-{u_{\text{w}}}^{\infty }) \end{array}$$ (16)

where,$v_{\text{n}}$ is the component of the pore fluid velocity in the direction of the outward normal to the surface, m/s. $k_{\text{s}}$ is the seepage coefficient, m/(s·Pa). $u_{\text{w}}$ is the current pore pressure at this point on the surface, Pa. ${u_{\text{w}}}^{\infty }$ is a reference pore pressure value, Pa.

This boundary condition permits only the outflow of pore fluid from the simulation domain and does not allow any inflow. When the pore pressure on the boundary is positive, the fluid outflow rate is proportional to the boundary pore pressure. When the pore pressure is negative, the outflow rate is set to zero. This relationship is described in Equation (17).


$$\begin{array}{c} v_{n}=0,u_{w}\leq 0 \end{array}$$ (17)

This boundary condition is a simplified approach to represent the macroscopic effect of pressure depletion. It assumes that the fluid outflow is driven by the pressure difference, effectively mimicking the low-pressure zone created by the parent well production, which is the key factor influencing stress reorientation.

Fig 3 illustrates the coupled calculation workflow of the model. First, the hydraulic fractures of the parent well are set as drainage boundaries to simulate the production process. The continuous extraction of reservoir fluid through these boundaries leads to a decrease in pore pressure in the near-wellbore region of the fractures, while the pressure in the far-field region remains unchanged, thereby generating a significant pore pressure gradient. Governed by the fluid flow module, fluid flows toward the fracture zone along this pressure gradient and is extracted, driving the dynamic reconstruction of the reservoir's pore pressure field. The variation in this pore pressure field superimposes on the initial in-situ stress field, inducing the redistribution of the effective stress field in the reservoir. Subsequently, based on the reconstructed effective stress field, the XFEM is employed to simulate the hydraulic fracturing process of the infill wells, thereby achieving a fluid-solid coupling analysis of the effective stress evolution induced by parent well production and the fracture propagation in infill wells.

**Fig 3:**
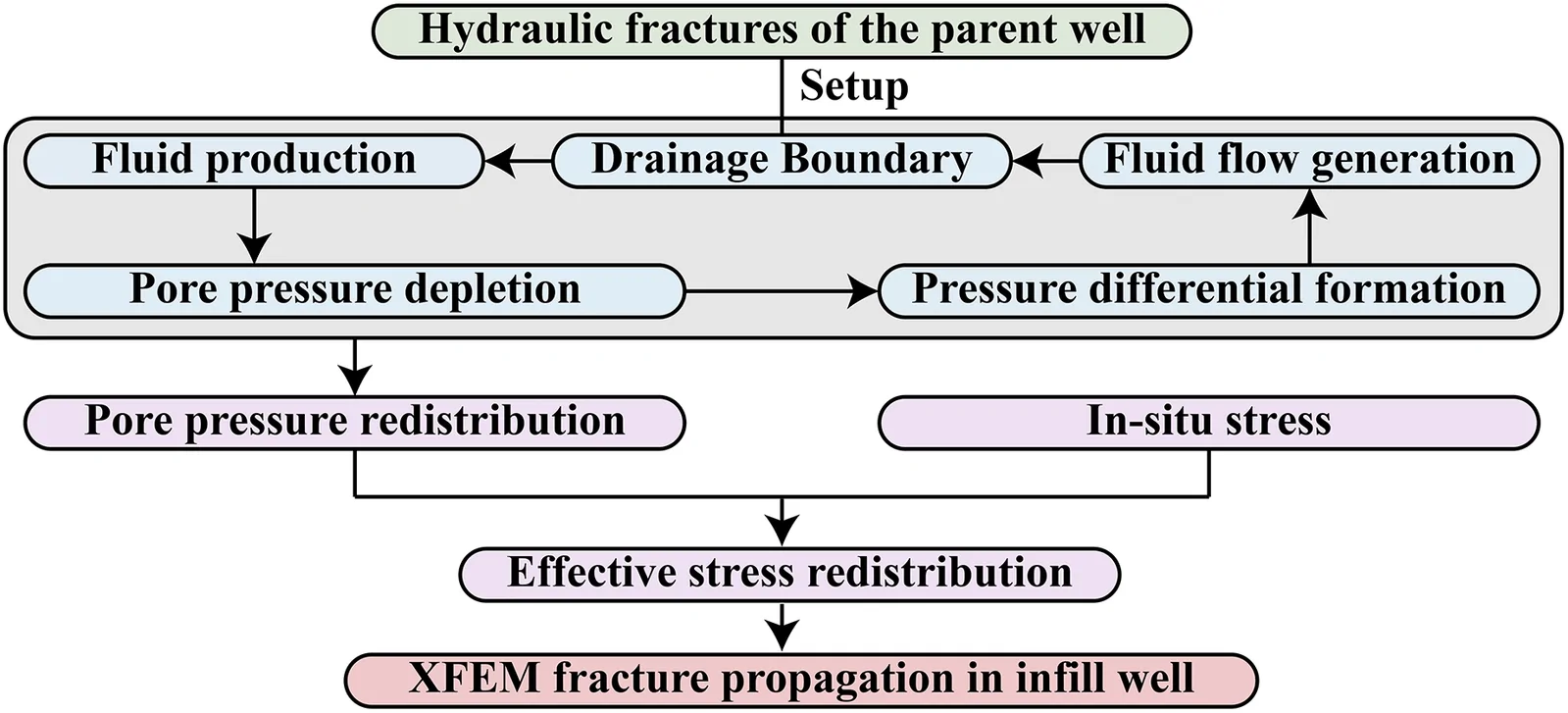
The coupled calculation workflow of the model.

## 3. Model validation

Due to the long duration of field operations, the difficulty in characterizing reservoir fracture morphology, and the confidentiality of engineering data, numerical simulation studies on infill well fracturing often struggle to obtain complete field data for direct validation. Consequently, previous similar studies frequently lack a model validation step [20,33,34]. To address this challenge, this study adopts a step-by-step validation strategy. The numerical model constructed herein primarily involves two core physical processes: (1) the evolution of the pore pressure field induced by production, simulated based on a drainage boundary; (2) fracture propagation under the reconstructed stress field, simulated using XFEM. Given that the application of XFEM in hydraulic fracturing simulation is highly mature, and its algorithmic accuracy has been extensively validated in numerous studies, it will not be reiterated here [35]. Therefore, this section focuses on verifying the accuracy of the free drainage boundary in simulating the reservoir production process. To achieve this, laboratory physical experiments were designed for comparative validation.

### 3.1 Experimental setup and design

Outcrop shale was selected as the experimental sample. This rock is characterized by high density, uniform texture, and the absence of apparent natural fractures, which effectively eliminates the interference of heterogeneity on the validation results. Testing results indicate that the rock has an elastic modulus of 21.63 GPa, a Poisson's ratio of 0.2, a porosity of 4%, and a permeability of 5.92 × 10^-17^ m^2^.

The schematic diagram of the experimental apparatus is shown in Fig 4. The core component is a self-developed dual-port high-pressure sealed container. One port is connected to a high-pressure gas source to apply pore pressure, thereby simulating the original formation pressure. The other port is connected to a simulated wellbore inside the rock sample. Coupled with a constant-pressure back pressure regulator to control the outlet pressure, this setup simulates the production process of oil and gas extraction from the wellbore. Additionally, a flow meter and a gas recovery container are connected in series at the end of the pipeline to monitor and record the production data in real time.

**Fig 4:**
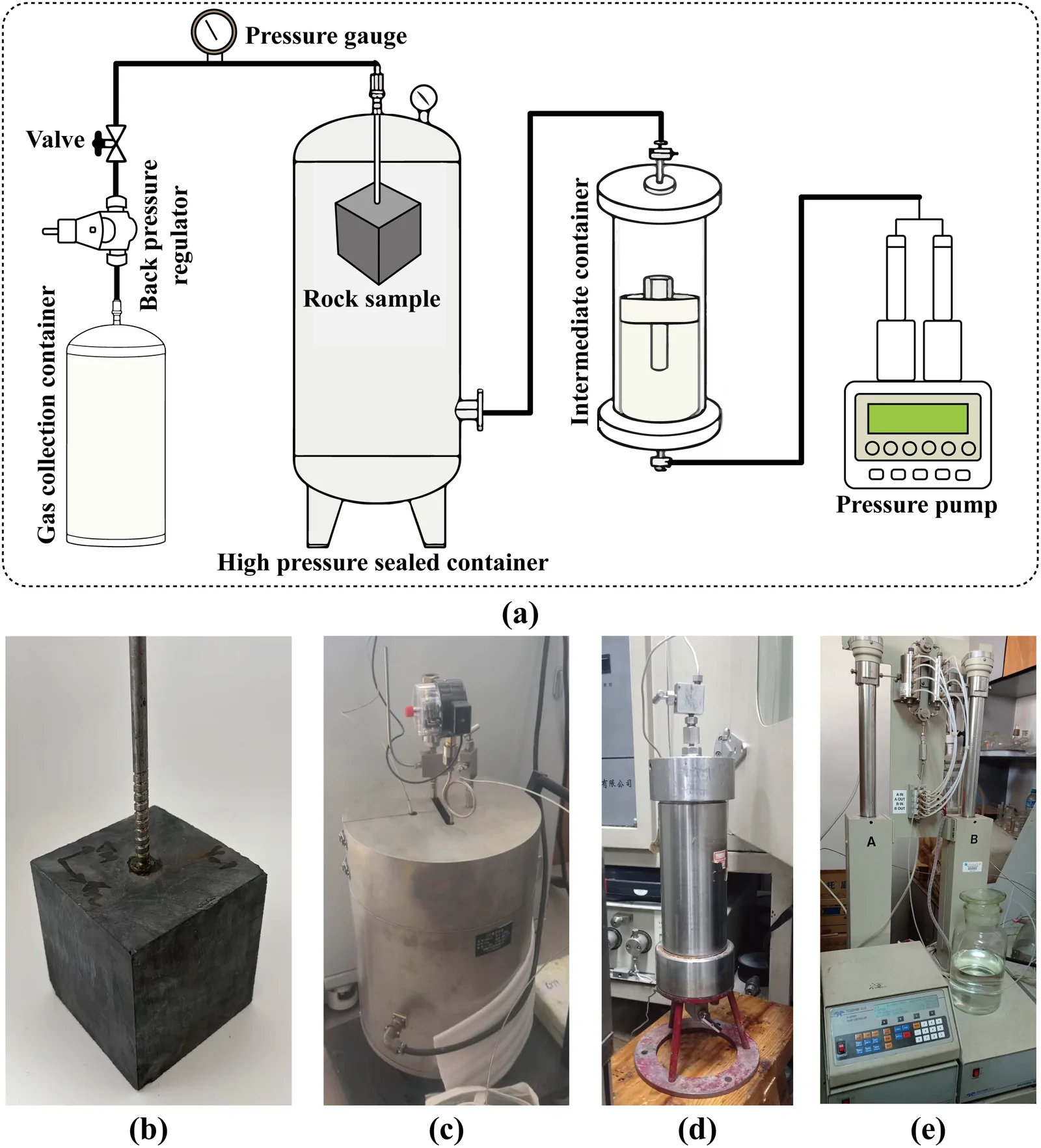
Experimental apparatus.

The experiment was designed to simulate the shale gas production process. The specific procedures are as follows:

(1) Sample preparation: The outcrop shale was machined into a standard cubic sample with an edge length of 100 mm.
(2) Wellbore prefabrication: A wellbore with a diameter of 10 mm and a depth of 55 mm was drilled vertically downward from the center of the top surface of the sample.
(3) Casing and cementing: A hollow metal tube was fabricated to serve as a simulated casing. It was run into the wellbore to a depth of 45 mm and cemented in place using epoxy resin. A 10-mm open-hole section was intentionally left at the bottom to simulate the production zone.
(4) Apparatus assembly: The rock sample was placed inside the high-pressure vessel. The bottom port of the vessel was connected to a constant-pressure pump to apply the formation pressure, while the top port was connected to the upper end of the simulated wellbore. The connection configuration is illustrated in Fig 4.
(5) Saturation and pressurization: With the valves closed, shale gas was injected into the vessel through the bottom port. The pressure gauge was increased to 20 MPa and maintained steadily to simulate the original formation pressure condition.
(6) Constant-pressure production: The constant-pressure back-pressure valve was adjusted to 5 MPa, and the valve was opened to simulate the constant-pressure production process under a bottom-hole flowing pressure of 5 MPa. The production data were recorded via a flow meter.

### 3.2 Model validation results

A corresponding numerical model was established based on the aforementioned experimental conditions, with the rock property parameters identical to those listed in Table 1. The boundary conditions of the model were configured as follows: the outer surface of the rock sample was set as a constant-pressure boundary (20 MPa), and the open-hole section was defined as a drainage boundary (5 MPa). The fluid parameters of shale gas under different pressures are presented in Table 1.

**Table 1:** Physical property parameters of shale gas.

Pressure condition	20MPa	5MPa
Density (kg/m^3^)	150	35
Viscosity (Pa/s)	1.5 × 10^−10^	1.1 × 10^−10^

The simulation results are presented in Fig 5. By sectioning the model along the plane illustrated in Fig 5(a), Fig 5(b) displays the pore pressure distribution on the cross-section, while Fig 5(c) shows the characteristics of the flow velocity vectors. As can be seen from the figure, the pore pressure exhibits a distinct decreasing gradient, which is consistent with the fundamental physical laws of fluid seepage. The flow velocity vectors indicate that the fluid continuously converges from the exterior of the sample toward the open-hole section and is ultimately extracted through it. Meanwhile, no fluid outflow is observed in other regions where no drainage boundary is defined. This simulation result accurately reproduces the fluid production characteristics observed in the experiment.

**Fig 5:**
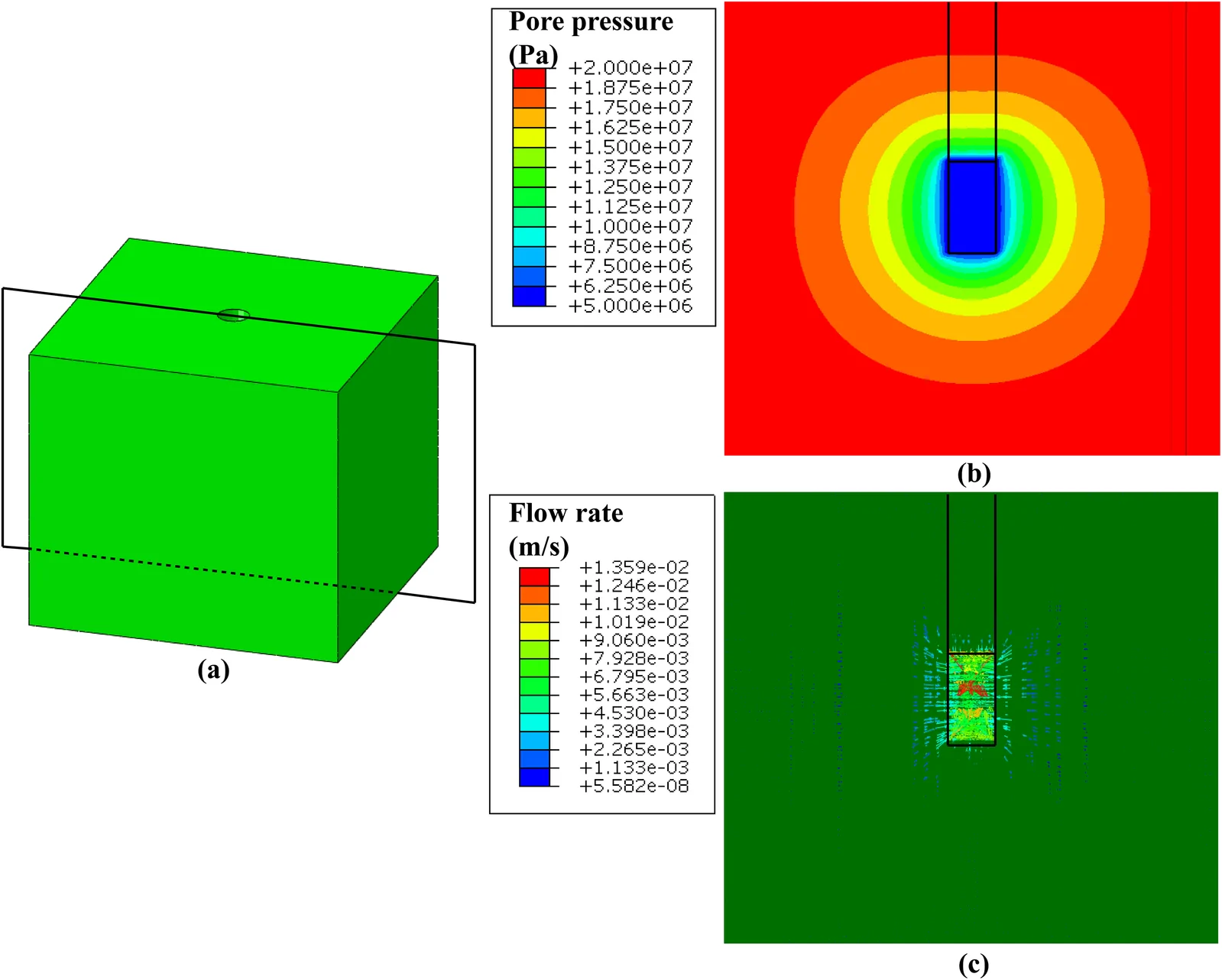
Simulation results of the verify model.

Model calculations indicate that when the flow reaches a steady state, the theoretical flow rate under the 20 MPa condition is 4.439 mL/s. When converted to the experimental acquisition environment (5 MPa), the corresponding standard flow rate is 19.02 mL/s. Comparing this with the experimental data, when the physical experiment reaches the stable production stage, the measured flow rate is 17.37 mL/s. The relative error between the numerical simulation results and the experimental data is merely 9.5%. Considering the extreme randomness of natural rock cores and the systematic errors inherent in the experimental system, this error margin is well within an acceptable range. This result fully demonstrates the reliability and accuracy of employing the free drainage boundary to simulate the reservoir production process and pressure depletion.

## 4. Results and analysis

The Eagle Ford Shale is a representative low-permeability unconventional reservoir. After primary fracturing, the reservoir pressure declines rapidly and the stable production period is short, necessitating infill fracturing operations to further recover hydrocarbons from the interwell region of mature wells. In this study, the Eagle Ford Shale in the United States [12] is selected as the target formation for numerical simulation of fracture propagation during infill fracturing. The basic parameters are listed in Table 2 [33]. The specific model setup is shown in Fig 6, two previously fractured parent wells are placed on either side of the infill well, with a well spacing of 400 m. The parent wells were developed using a multi-cluster, staged hydraulic fracturing approach, with fracture half-lengths ranging from 50 m (minimum) to 150 m (maximum), and a cluster spacing of 23 m. The infill well is also fractured using a perforation-based method, with a fracture spacing of 20 m. Due to long-term production from the parent wells, a significant decrease in pore pressure has occurred; therefore, the hydraulic fractures in the parent wells are assigned free-drainage boundary conditions. After a period of production from the parent wells, infill fracturing is performed, followed by analysis of how different operational conditions affect fracture propagation in the infill well.

**Table 2:** Basic parameters for infill fracturing numerical simulation.

Parameter	value	Parameter	value
Matrix permeability (m^2^)	4.6 × 10^–16^	Matrix porosity (/)	0.12
Initial reservoir pressure (MPa)	56.02	Elastic modulus (GPa)	20.0
Poisson's ratio (/)	0.22	Tensile strength (MPa)	10.0
Injection rate (m^3^/min)	15	Perforation angle (°)	90
Operation time (s)	7200	Maximum principal stress (MPa)	75
Reservoir thickness (m)	23	Parent well pressure (MPa)	20.7
Minimum principal stress (MPa)	65	Vertical stress (MPa)	68
Leak-off coefficient (m/s)	1 × 10^–12^	Fluid viscosity (Pa·s)	1 × 10^−5^
Biot coefficient	0.7		

**Fig 6:**
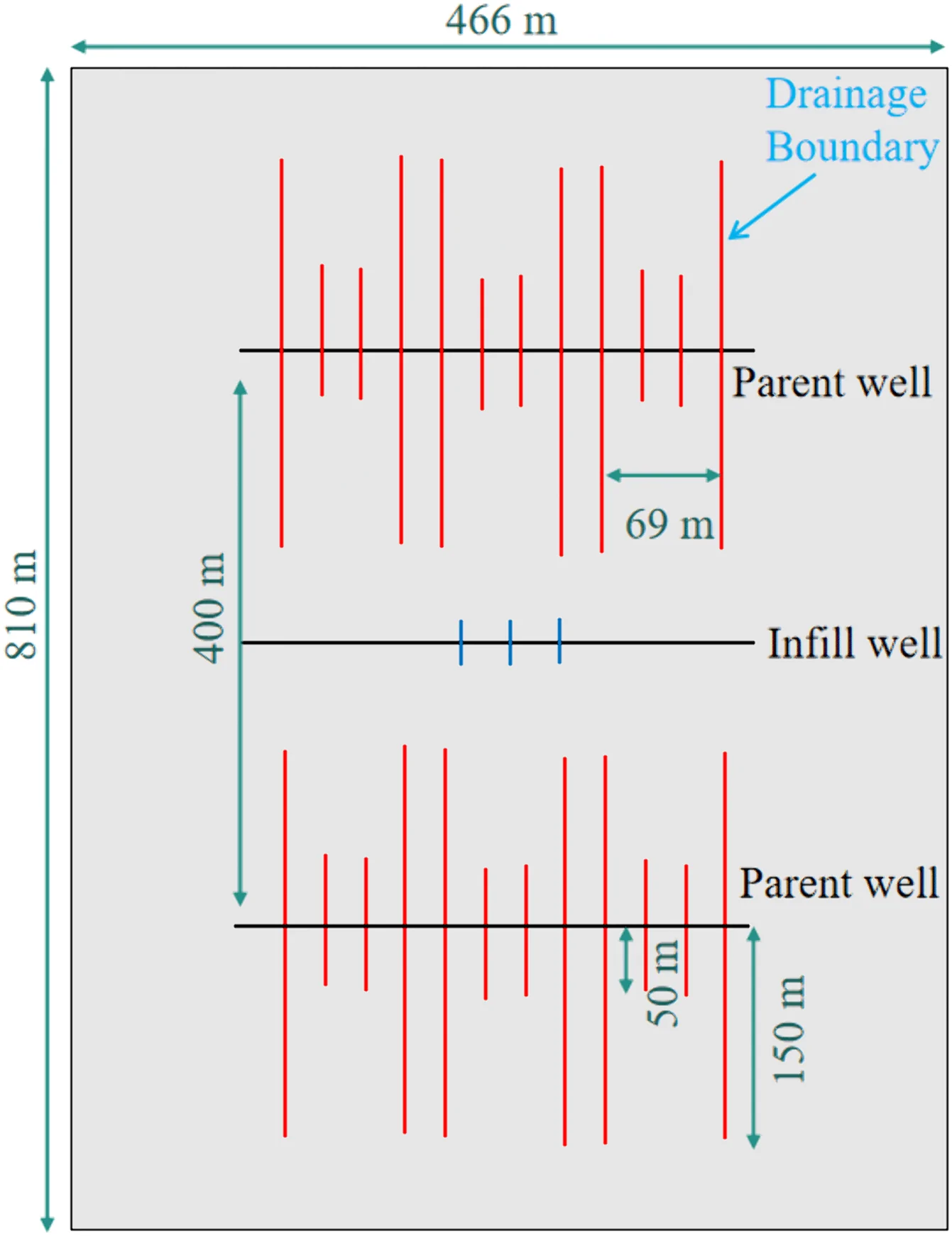
Basic model for infill fracturing.

To evaluate the post-fracturing fracture propagation results, this study considers not only the fracture length—which is commonly regarded as a key indicator of stimulation effectiveness, with longer fractures generally implying better performance [36]—but also the fracture tortuosity to characterize the fracture trajectory after infill fracturing. For simple fractures, the tortuosity is defined as the ratio of the horizontal to vertical distance between the fracture tip and the initiation point. For highly curved fractures in which the tip passes the initiation point multiple times, tortuosity is quantified as the sum of the absolute horizontal deviations along the x-direction (Fig 7). This definition is essentially a simplified form of the rise-to-span ratio, a fundamental geometric concept widely used in engineering to describe the curvature of slender structures, it is geometrically equivalent to a first-order approximation of tortuosity.

**Fig 7:**
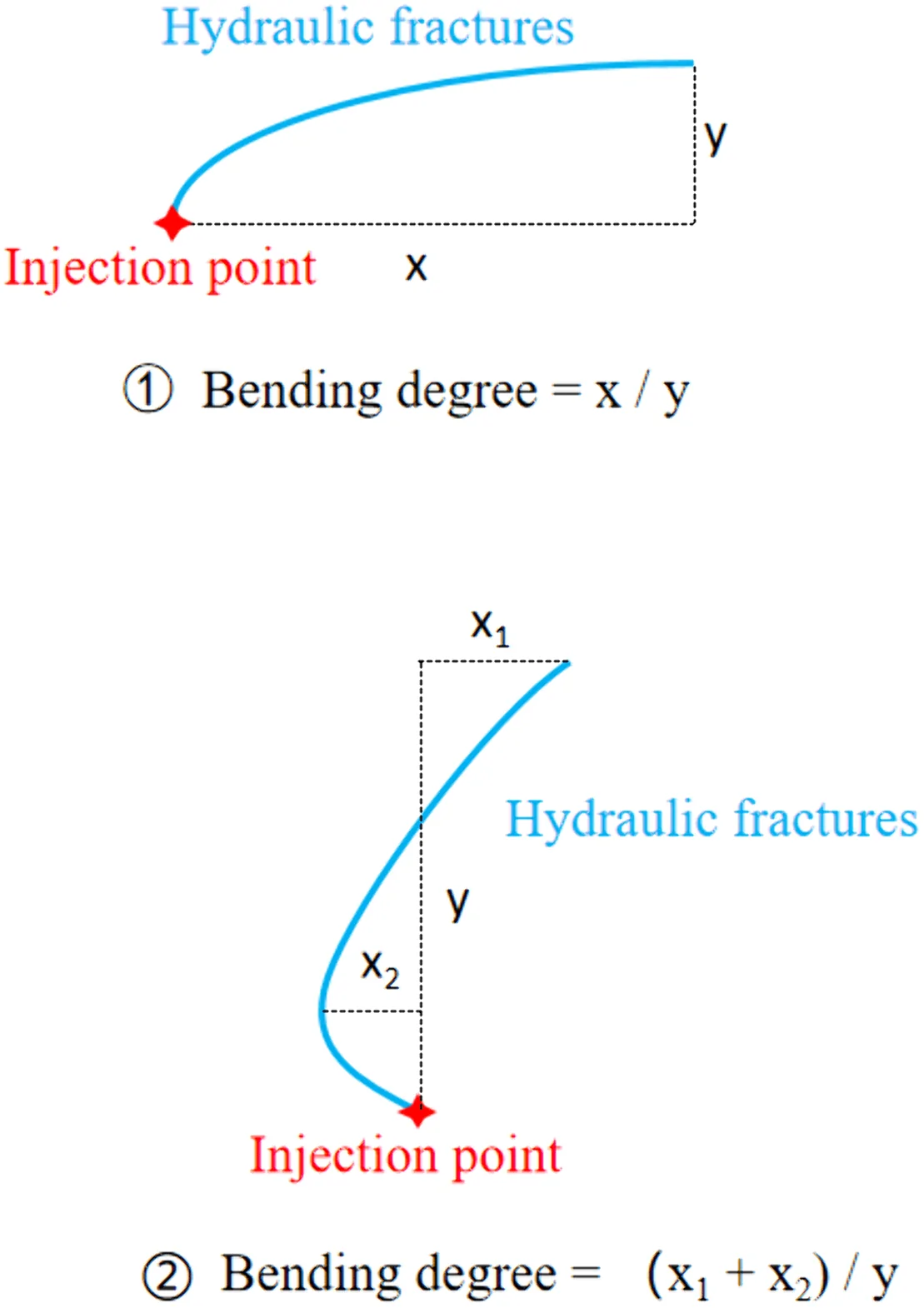
Method for calculating fracture bending degree.

The numerical simulations were performed using ABAQUS. A structured mesh with a minimum element size of 3 m around the wellbore was adopted. A mesh convergence test confirmed that further refinement did not significantly alter the fracture trajectory. The maximum time increment was set to 10 s to ensure convergence.

### 4.1 Comparison between infill fracturing and conventional fracturing

Fracture propagation in infill wells at different production times was simulated and compared with that of conventional fracturing, as shown in Fig 8. The initial pore pressure was set to 56.02 MPa. In the conventional fracturing case, it is assumed that the reservoir fluid has not been depleted, and the fracture geometry is primarily influenced by inter-fracture stress shadow effects. At the early stage of propagation (1800 s), all three fractures initiate simultaneously with similar lengths, and they propagate predominantly along the direction of maximum horizontal principal stress. As injection time increases, the fractures extend toward the far field. To maintain propagation, the internal fluid pressure within the fractures rises, intensifying the interference between adjacent fractures. At this stage, the propagation direction of the middle fracture remains largely unchanged, while the two outer fractures symmetrically extend toward the direction of minimum horizontal principal stress. In the case of infill fracturing, the injected fluid serves to propagate the fracture and maintain its width; however, the low-pore-pressure region associated with the parent well disrupts this process through coupled mechanisms. Specifically, the pressure differential diverts fluid into the depleted zone to replenish reservoir energy, which diminishes the fluid volume available for propagation. Simultaneously, this pore pressure depletion alters the effective stress state and, based on poroelastic theory, triggers a redistribution of the in-situ stress field. These factors collectively limit the fluid efficiency and alter the stress conditions, ultimately constraining the fracture's further propagation. Under the simulation parameters of this study, after 7200 seconds, the fracture length in the conventional fracturing case is nearly 30 meters longer than that in the infill fracturing case. The influence of the low-pore-pressure region around the parent well on fracture propagation is also reflected in fracture geometry. In conventional fracturing, the fracture morphology is relatively regular, with a bending degree of only 1.86. In contrast, fracture propagation during infill fracturing is significantly affected by the heterogeneous pore pressure distribution in the far-field reservoir, resulting in a pronounced increase in fracture curvature. Under the same simulation time, the bending degree of infill fractures is approximately 30% higher than that of conventional fractures.

**Fig 8:**
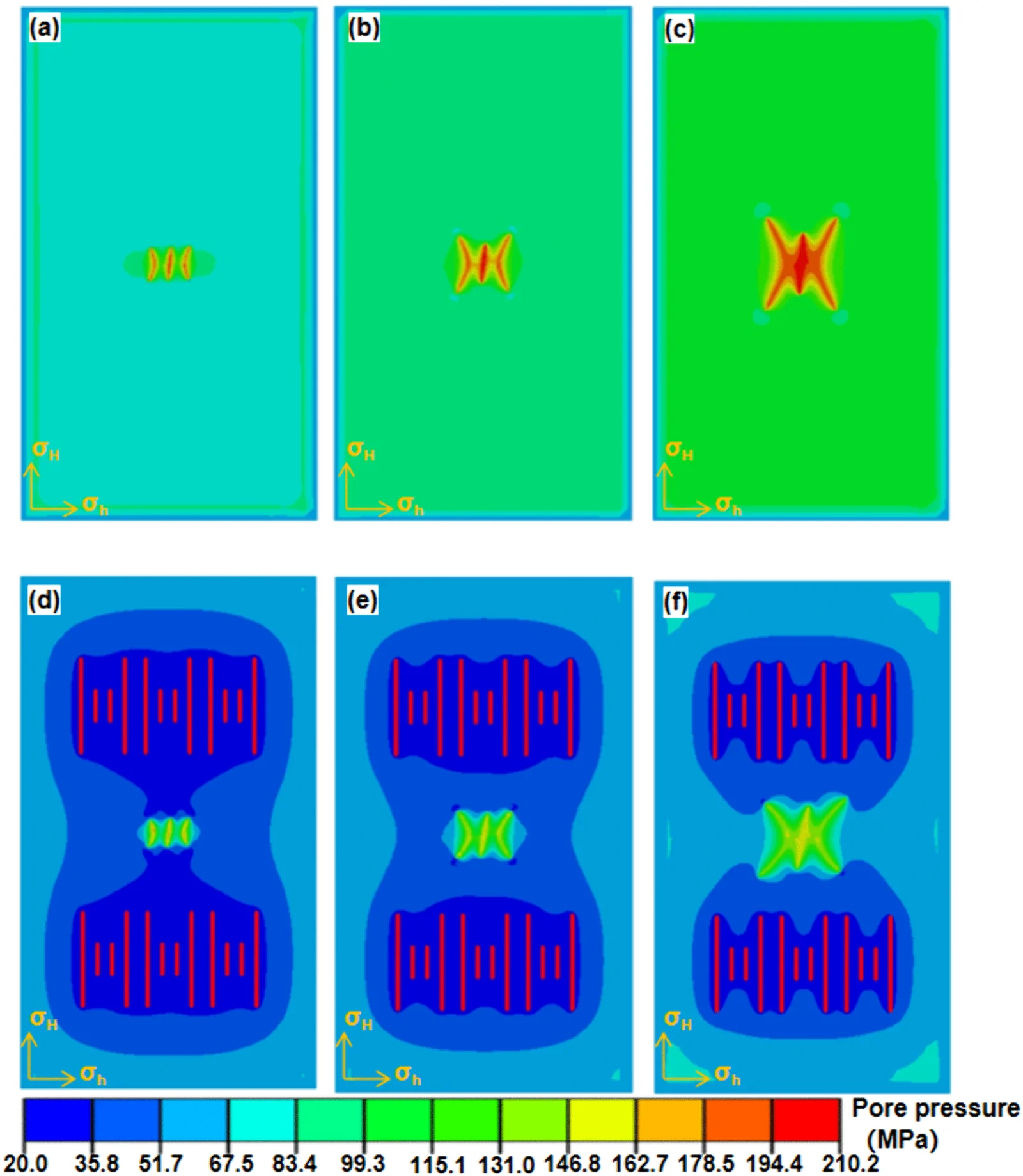
Fracture propagation trajectories at different times: (a) conventional fracturing – 1800 s; (b) conventional fracturing – 3600 s; (c) conventional fracturing – 7200 s; (d) infill fracturing – 1800 s; (e) infill fracturing – 3600 s; (f) infill fracturing – 7200 s.

### 4.2 Influence of parent well production on fracture propagation in infill fracturing

As reservoir production time increases, fluids within the formation are continuously extracted, resulting in a decline in reservoir energy. The resulting depletion zones alter the original in-situ stress distribution. In this study, a free-drainage boundary module is employed to simulate the production process of the parent well, with production durations set to 1 year, 3 years, and 5 years, respectively, followed by infill fracturing simulations. The results are shown in Fig 9. As production time increases, the pore pressure near the parent well fractures decreases significantly, with the lowest pore pressure reaching approximately 20 MPa after 5 years of production. In terms of pore pressure distribution, the low-pressure region is mainly concentrated around the parent well fractures, which reflects a typical characteristic of low-permeability reservoir development: once beyond the effective range of hydraulic fracturing, it becomes difficult to efficiently extract hydrocarbons from the far-field region. After 1 year of production, the fracture geometry resulting from infill fracturing is similar to that of conventional fracturing, with the central fracture propagating nearly parallel to the maximum horizontal principal stress. However, as production time increases and pore pressure around the parent well significantly declines, the fracture propagation behavior is noticeably altered, leading to greater fracture deflection. Fracture parameter statistics (Fig 10) indicate that, under identical operational conditions, the timing of infill fracturing has no significant effect on fracture propagation length—the total fracture length in all three simulation cases is approximately 380 m. As production time increases, the fracture bending degree shows an upward trend. When the parent well production time is extended from 1 year to 3 years, the tortuosity increases from 2.41 to 2.58, representing a 7.1% increase. Furthermore, when extended to 5 years, the tortuosity rises to 3.18, marking a substantial increase of 23.8%. It is worth noting that while a higher bending degree indicates a larger contact area with the reservoir, excessive tortuosity may increase flow resistance and hinder proppant transport. Therefore, an optimal fracture geometry should balance complexity and conductivity.

**Fig 9:**
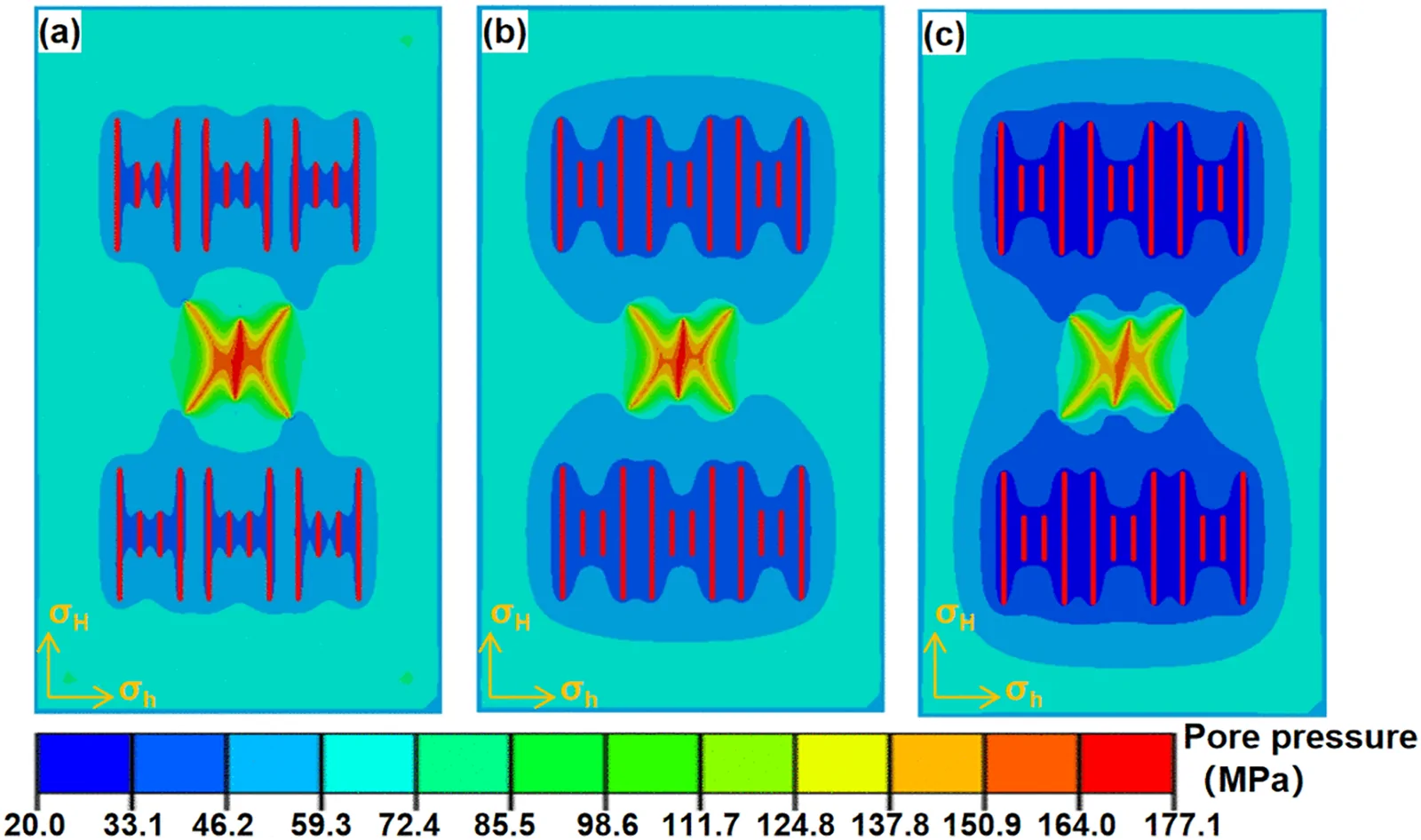
Fracture propagation trajectories of the infill well after different parent well production times: (a) 1 year; (b) 3 years; (c) 5 years.

**Fig 10:**
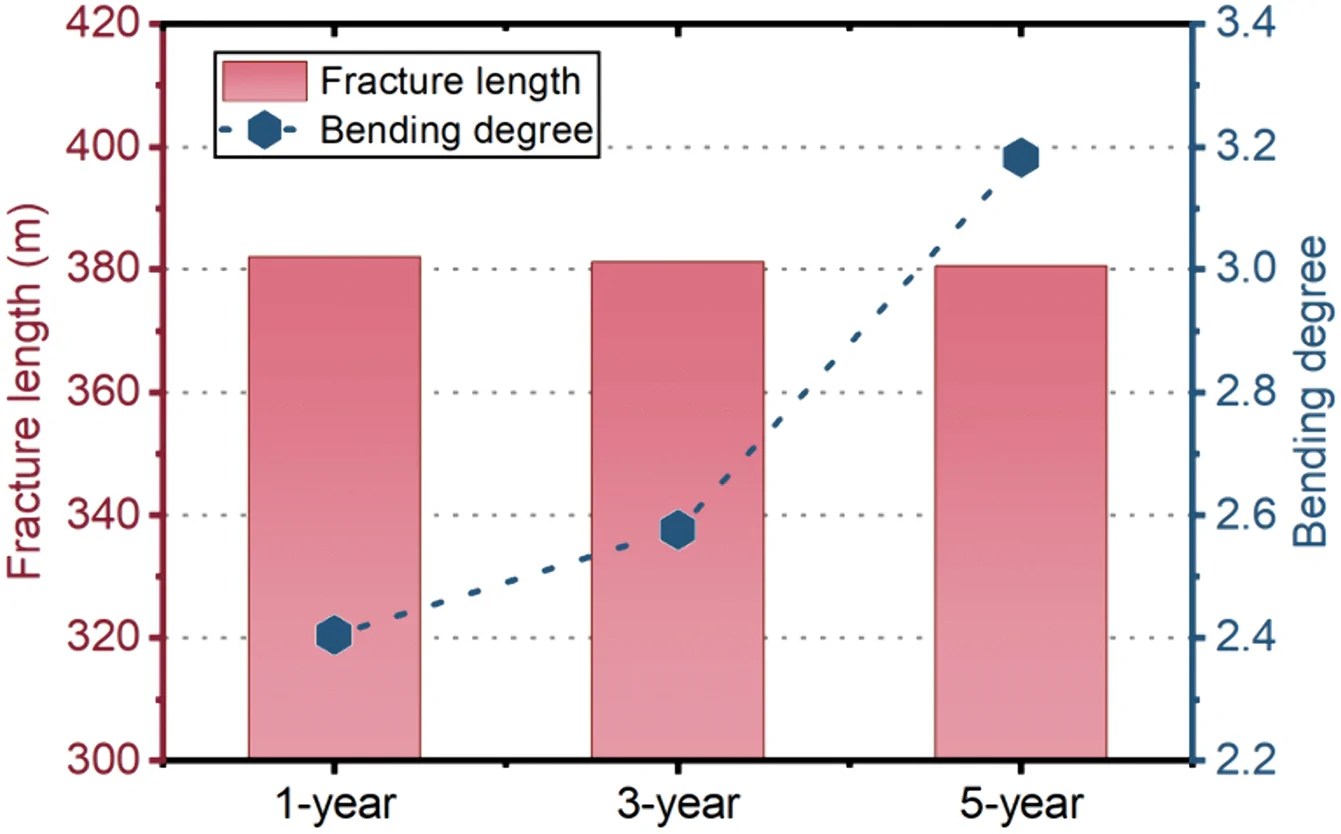
Fracture parameter statistics of the infill well after different parent well production times.

### 4.3 Influence of injection rate on fracture propagation in infill fracturing

Injection rate is one of the key fracturing parameters in the development of unconventional reservoirs. In this study, infill well injection rates were set at 10 m^3^/min, 15 m^3^/min, and 20 m^3^/min to simulate infill fracturing after 5 years of parent well production. The resulting fracture propagation patterns are shown in Fig 11. Fracture length exhibits a trend of initially increasing rapidly and then slowing down as the injection rate rises. Specifically, when the injection rate increases from 10 m^3^/min to 15 m^3^/min, the fracture length grows from 364.7 m to 380.7 m, representing an increase of 4.38%. However, as the injection rate further increases from 15 m^3^/min to 20 m^3^/min, the fracture length reaches 383.9 m, with a marginal increase of 1.67%. In terms of fracture geometry, the case with an injection rate of 10 m^3^/min exhibits a relatively limited propagation range, with greater deflection observed in the fracture trajectory toward the far-field region. As the injection rate increases, the fluid within the fractures carries greater energy, reducing the influence of the stress field induced by prior parent well fracturing. Consequently, the fracture length increases, and the deflection at the fracture tip decreases. At an injection rate of 10 m^3^/min, the fracture bending degree is 3.95, while increasing the rate to 20 m^3^/min reduces the bending degree to 2.65 (Fig 12). In practical infill fracturing operations, if the goal is to generate many complex fractures near the wellbore to primarily extract hydrocarbons from the near-well region, a lower injection rate is recommended. Conversely, to accelerate fracture propagation and promote connectivity with existing parent well fractures, a higher injection rate should be adopted to mitigate the stress interference effect from the parent well.

**Fig 11:**
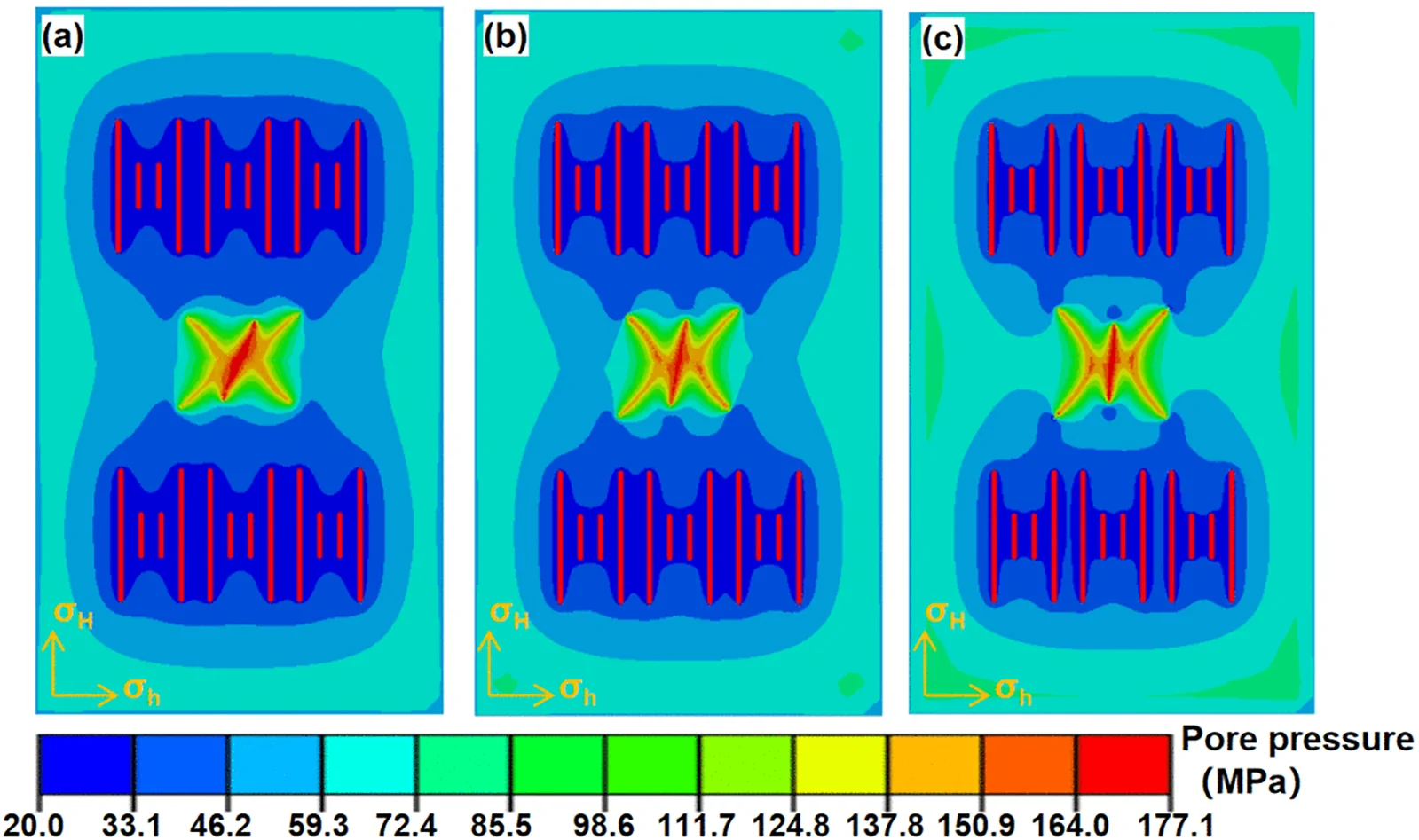
Fracture propagation trajectories of the infill well under different injection rates: (a) 10 m^3^/min; (b) 15 m^3^/min; (c) 20 m^3^/min.

**Fig 12:**
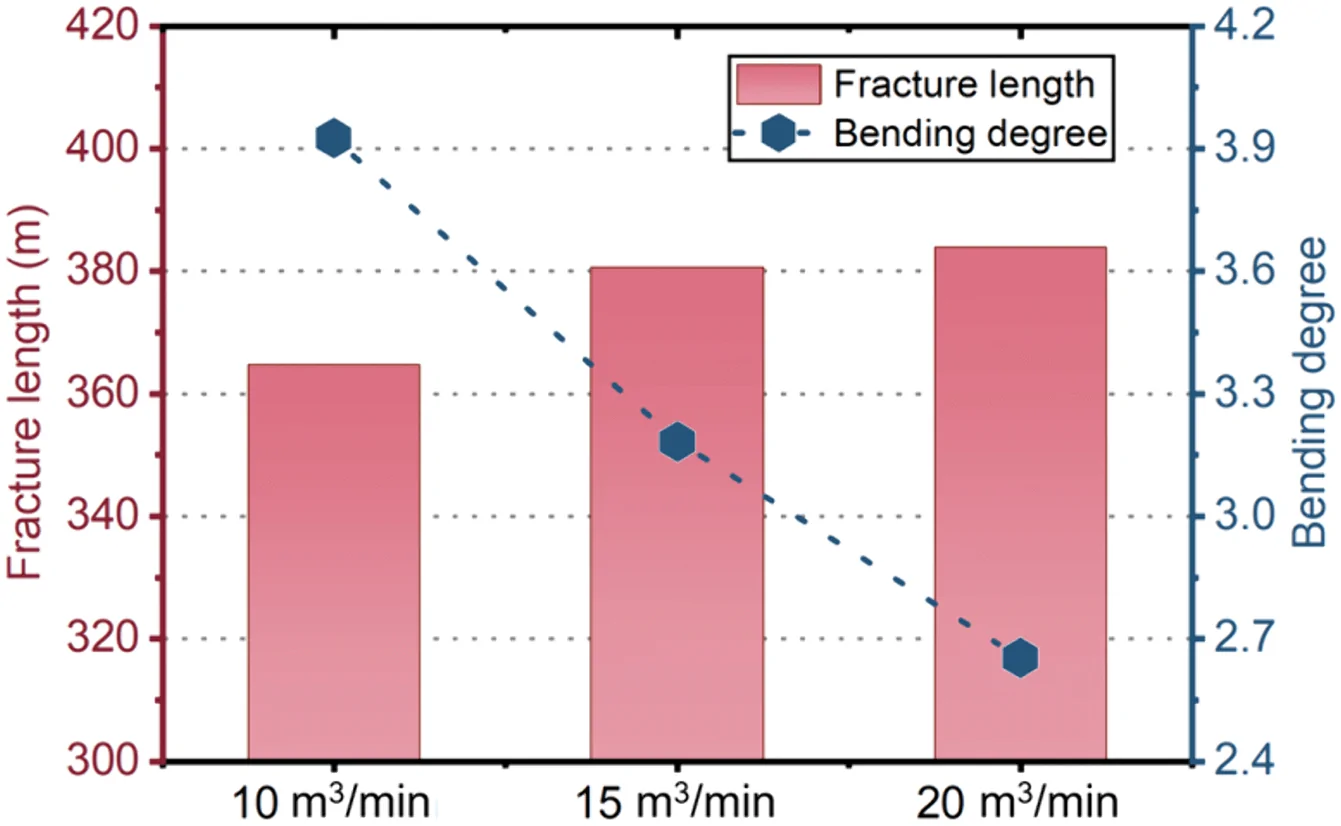
Fracture parameter statistics of the infill well under different injection rates.

### 4.4 Influence of perforation orientation on fracture propagation in infill fracturing

Perforation angles (i.e., the angle between the perforation direction and the wellbore axis) were set to 30°, 60°, and 90° to analyze their influence on fracture propagation during infill fracturing. The simulation results are shown in Fig 13. As the perforation angle increases, the fracture deflection becomes more pronounced. When the perforation azimuth is 30°, the fracture bending degree is 4.33 (Fig 14). As the perforation angle increases to 90°, the bending degree decreases by 41.8%. Meanwhile, the fracture length first increases and then decreases, the fracture length reaches its maximum at a perforation angle of 60°. Compared to the cases at 30° and 90°, the fracture length increases by 1.5% and 6.62%, respectively. Previous studies have shown that [37], under a given in-situ stress condition, the perforation angle affects the distribution of shear and tensile stresses at the fracture tip, and selecting a smaller perforation azimuth is generally favorable for enhancing hydraulic fracturing effectiveness. For infill fracturing, under the parameters used in this study, a perforation angle of 60° yields the maximum fracture length with a moderate bending degree, which is more beneficial for improving reservoir stimulation performance.

**Fig 13:**
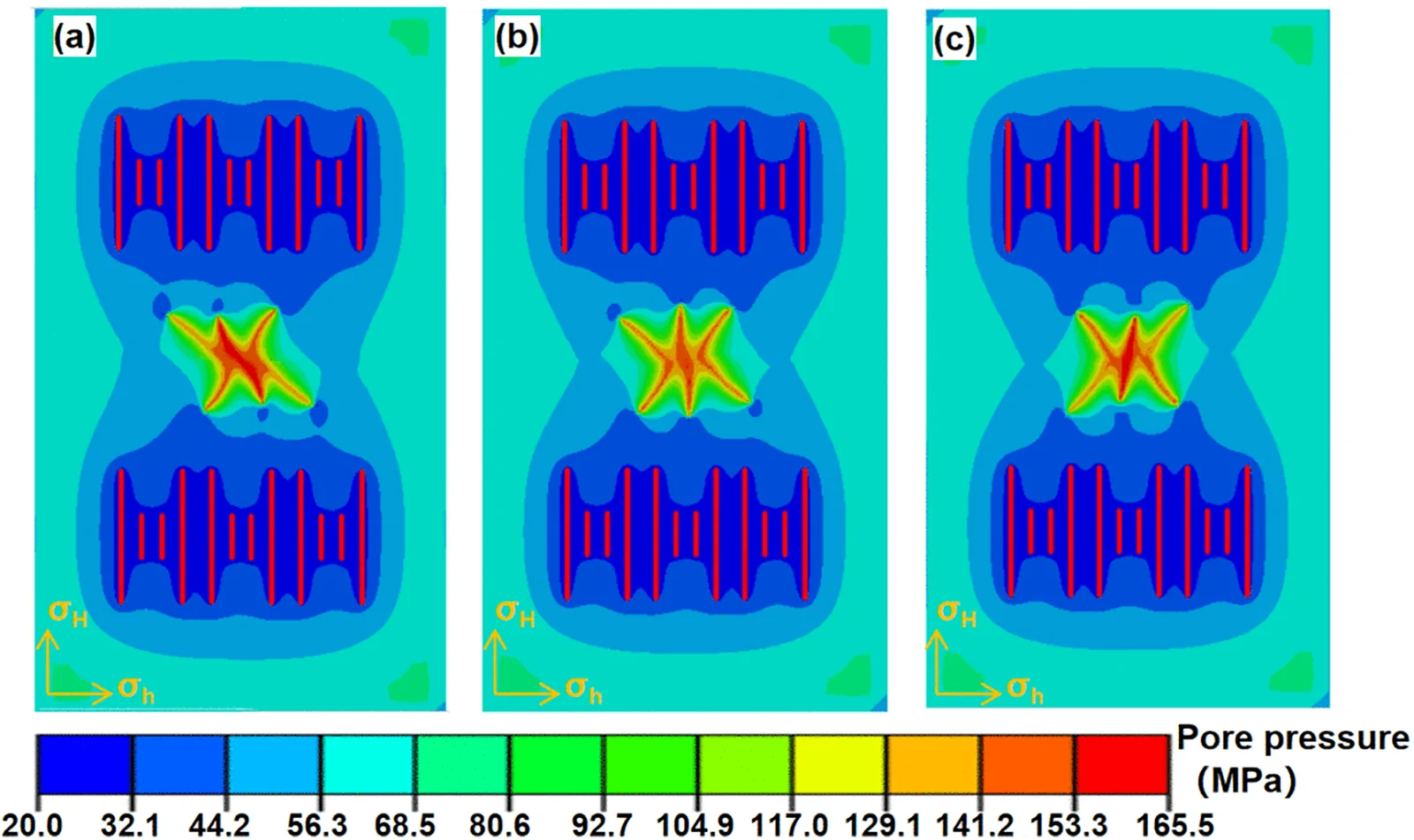
Fracture propagation trajectories of the infill well under different perforation angles: (a) 30°; (b) 60°; (c) 90°.

**Fig 14:**
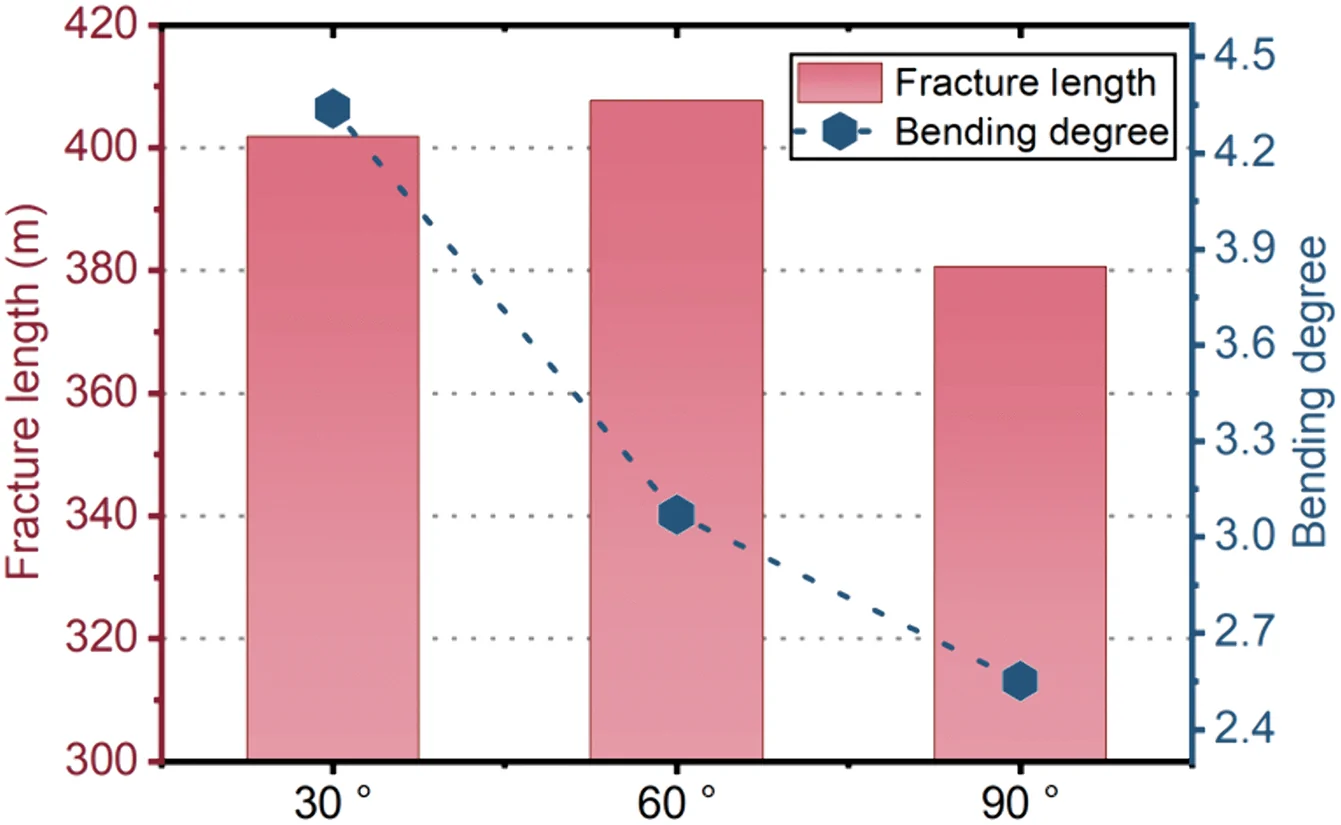
Fracture parameter statistics of the infill well under different perforation angles.

## 5. Discussion

In this work, a numerical model for mature well infill fracturing was developed based on the XFEM and a free-drainage boundary module. The influence of various parameters on fracture propagation in infill wells was analyzed. The findings of this study provide meaningful guidance for the design of construction parameters in infill fracturing operations. However, this study still has several limitations that warrant further improvement.

First, this study adopts a homogeneous reservoir assumption, which inherently differs from the strong heterogeneity and anisotropy typically present in subsurface oil and gas reservoirs. However, this simplification is deliberately made to align with the specific research objectives of this paper. The core aim of this study is to investigate the direct regulatory effects of artificially controllable engineering parameters on fracturing outcomes, rather than focusing on the inherent reservoir properties that serve as geological constraints. In engineering practice, reservoir physical parameters are generally treated as predetermined conditions, while engineers are more concerned with optimizing design variables to improve development performance. Therefore, through deterministic sensitivity analysis, this study highlights the fracture propagation laws under specific reservoir conditions. Admittedly, the randomness of natural properties can indeed exert a significant impact on engineering outcomes. In light of this, future research will build upon the current work by incorporating stochastic rock physics properties to conduct comprehensive uncertainty quantification studies, including Monte Carlo simulations. This will further bridge the gap between idealized models and complex geological conditions.

Moreover, natural fractures play a pivotal role in enhancing fracture network complexity; however, their quantitative characterization remains a significant challenge. To isolate and clearly investigate the specific effects of free-drainage boundaries and pore pressure depletion on fracture propagation, this study adopted a simplified homogeneous model. Introducing complex natural fracture networks and anisotropy at this stage would introduce excessive variables, potentially obscuring the primary mechanisms governing the parent-well interaction. Consequently, the current model assumes a continuum without pre-existing joints to ensure numerical convergence and focus on the hydraulic fracture trajectory in intact rock. We acknowledge, however, that neglecting natural fractures limits the direct applicability of these results to naturally fractured formations, such as coal seams or jointed rock masses. Therefore, future research will extend this framework to incorporate stochastic discrete fracture networks, aiming to provide a more comprehensive understanding of fracture propagation in complex geological settings. Finally, current research primarily focuses on fracture length and morphology. Future studies will couple these fracture models with the production process to analyze well productivity under different fracture geometries.

## 6. Conclusion

(1) A numerical model for mature well infill fracturing was developed based on the extended finite element method (XFEM) and a free-drainage boundary module. This model enables the analysis of dynamic pore pressure evolution under different production durations and allows investigation of how the altered in-situ stress field influences fracture morphology in infill wells.
(2) Using the reservoir conditions of the Eagle Ford shale as a case study, the impact of parent well production on infill fracturing is mainly reflected in fracture length and bending degree. Under conventional fracturing, fracture geometry is relatively regular, with a bending degree of only 1.86. In contrast, fracture propagation in infill fracturing is significantly influenced by the heterogeneous pore pressure field near the parent well, resulting in a bending degree approximately 30% higher and a shorter fracture length under the same simulation time.
(3) As production time increases, fracture tortuosity in the infill well tends to rise. This leads to more extensive contact between the fractures and the reservoir. Under the Eagle Ford shale reservoir conditions and the operational parameters used in this study, performing infill fracturing after long-term parent well production is more beneficial for mobilizing far-field hydrocarbon resources.
(4) Increasing the perforation angle leads to greater fracture deflection, while the fracture length first increases and then decreases. Lower injection rates result in higher fracture bending degrees, whereas higher rates effectively increase fracture length. The injection rate for infill fracturing should be reasonably selected based on development objectives.
